# Papel Incremental da Classificação da New York Heart Association e dos Índices do Teste de Exercício Cardiopulmonar para Prognóstico na Insuficiência Cardíaca: um Estudo de Coorte

**DOI:** 10.36660/abc.20230077

**Published:** 2023-12-06

**Authors:** Pedro Henrique de Borba Engster, André Zimerman, Thomas Schaan, Marina S. Borges, Gabriel Souza, Giovanni Donelli Costa, Luis Eduardo Rohde, Anderson Donelli da Silveira

**Affiliations:** 1 Universidade Federal do Rio Grande do Sul FAMED Porto Alegre RS Brasil Universidade Federal do Rio Grande do Sul – FAMED , Porto Alegre , RS – Brasil; 2 Hospital de Clínicas de Porto Alegre Porto Alegre RS Brasil Hospital de Clínicas de Porto Alegre , Porto Alegre , RS – Brasil; 3 Harvard Medical School Boston Massachusetts EUA Harvard Medical School , Boston , Massachusetts – EUA; 4 Hospital Moinhos de Vento Porto Alegre RS Brasil Hospital Moinhos de Vento – Cardiologia, Porto Alegre , RS – Brasil

**Keywords:** Insuficiência Cardíaca, Prognóstico, Teste de Esforço

## Abstract

**Fundamento:**

A precisão da classificação da New York Heart Association (NYHA) para avaliar o prognóstico pode ser limitada em comparação com os parâmetros objetivos do teste de exercício cardiopulmonar (TECP) na insuficiência cardíaca (IC).

**Objetivo:**

Investigar o valor prognóstico da classificação da NYHA e da classe Weber.

**Métodos:**

Foram incluídos pacientes ambulatoriais adultos com IC submetidos a TECP em um centro terciário brasileiro. A classe NYHA atribuída pelo médico e a classe Weber derivada do TECP foram estratificadas como “favorável” (NYHA I ou II com Weber A ou B) ou “adversa” (NYHA III ou IV com Weber C ou D). Pacientes com uma classe favorável e uma classe adversa foram definidos como “discordantes”. O desfecho primário foi o tempo para mortalidade por todas as causas. Um valor de p bilateral < 0,05 foi considerado estatisticamente significativo.

**Resultados:**

Foram incluídos 834 pacientes. A mediana de idade foi de 57 anos; 42% (351) eram do sexo feminino e a mediana da fração de ejeção do ventrículo esquerdo foi de 32%. Entre os pacientes com classes NYHA e Weber concordantes, aqueles com classes NYHA e Weber adversas tiveram mortalidade por todas as causas significativamente maior em comparação com aqueles com classes favoráveis ( *hazard ratio* [HR]: 5,65; intervalo de confiança de 95%: 3,38 a 9,42). Entre os pacientes com classes discordantes, não houve diferença significativa na mortalidade por todas as causas (HR: 1,38; intervalo de confiança de 95%: 0,82 a 2,34). No modelo multivariado, incrementos na classe NYHA (HR: 1,55 por aumento na classe; intervalo de confiança de 95%: 1,26 a 1,92) e reduções no VO _2_ pico (HR: 1,47 por diminuição de 3 ml/kg/min; intervalo de confiança de 95%: 1,28 a 1,70) previu significativamente a mortalidade.

**Conclusões:**

A classe NYHA atribuída pelo médico e as medidas objetivas do TECP fornecem informações prognósticas complementares para pacientes com IC.

## Graphical abstract

**Figura Central:**
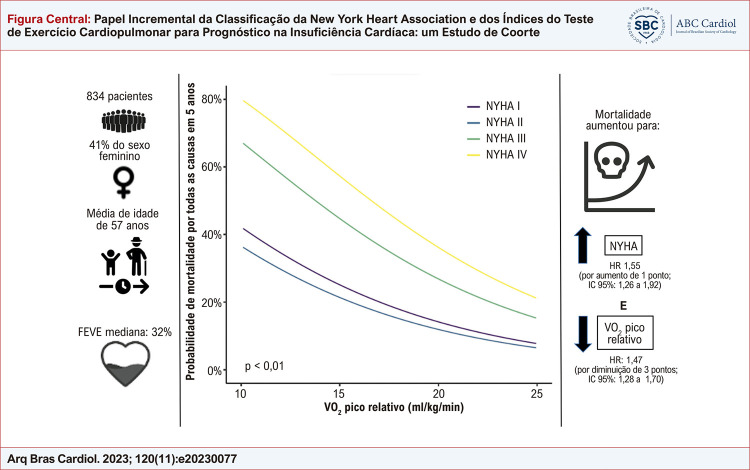
: Papel Incremental da Classificação da New York Heart Association e dos Índices do Teste de Exercício Cardiopulmonar para Prognóstico na Insuficiência Cardíaca: um Estudo de Coorte

FEVE: fração de ejeção do ventrículo esquerdo; HR: hazard ratio; IC: intervalo de confiança; NYHA: New York Heart Association; VO _2_: consumo de oxigênio.

## Introdução

A insuficiência cardíaca (IC) é uma das principais causas de morbidade e mortalidade ao redor do mundo, afetando mais de 64 milhões de pessoas. ^1^ Um dos pilares do manejo de pacientes com IC é a definição da classificação da New York Heart Association (NYHA), proposta em 1921 para medir o comprometimento funcional. ^2^ Essa medida subjetiva tem sido amplamente utilizada como critério de inclusão em protocolos clínicos. Pacientes considerados assintomáticos durante a atividade física normal (ou seja, classe I da NYHA) têm sido sistematicamente excluídos dos ensaios de IC. Consequentemente, as diretrizes clínicas frequentemente usam um ponto de corte da classe NYHA para determinar a elegibilidade para tratamentos como antagonistas dos receptores mineralocorticoides, inibidores do cotransportador sódio-glicose 2 e terapia de ressincronização cardíaca. ^3 - 5^ A classificação da NYHA é um preditor potente e estabelecido do prognóstico da IC em um nível de grupo. ^6 - 8^ Estudos recentes, entretanto, questionaram a reprodutibilidade da classificação da NYHA e sua capacidade de discriminar o prognóstico de pacientes com IC em nível individual. ^9 - 14^

Essas limitações têm estimulado esforços para obter parâmetros de capacidade funcional mais precisos e reprodutíveis em pacientes com IC, desde questionários estruturados até medidas objetivas de capacidade funcional, como o teste de exercício cardiopulmonar (TECP). ^10 , 15^ O TECP é um método não invasivo para analisar a aptidão cardiopulmonar e estabelecer o estado funcional. Atualmente, o TECP é utilizado para avaliar a gravidade da IC, monitorar a progressão da doença e determinar a elegibilidade para transplante cardíaco. ^16 - 18^

Para pacientes com IC, tanto a classe NYHA atribuída pelo médico quanto os parâmetros objetivos do TECP demonstraram ser fatores prognósticos independentes. ^7 , 19 , 20^ No entanto, como dois substitutos da capacidade funcional, um subjetivo e um objetivo, sua capacidade combinada de prognóstico é menos clara. Por exemplo, para pacientes submetidos a TECP, é plausível que a classificação da NYHA não tenha valor prognóstico adicional. Na presente análise, investigamos a interação dos índices derivados do TECP e da classe NYHA para refinar a avaliação prognóstica de pacientes com IC, particularmente quando o TECP e a classe NYHA apresentaram resultados conflitantes.

## Métodos

### Pacientes e desenho do estudo

O presente estudo de coorte incluiu pacientes consecutivos com IC que foram submetidos a TECP em um hospital terciário no Brasil entre janeiro de 2008 e novembro de 2020. O primeiro TECP de cada paciente foi incluído nesta análise. A classe da NYHA foi determinada imediatamente antes do TECP ou na consulta ambulatorial anterior. Os pacientes elegíveis tinham 16 anos ou mais, com IC documentada, diagnosticada por critérios clínicos, laboratoriais e ecocardiográficos. ^3^ Os pacientes deveriam estar clinicamente estáveis antes do TECP e usar terapia médica otimizada. Não houve critérios de elegibilidade relacionados à fração de ejeção do ventrículo esquerdo (FEVE), ou seja, pacientes com FEVE reduzida, levemente reduzida e preservada foram elegíveis para inscrição. Foram excluídos os pacientes que não conseguiram realizar o TECP. O presente estudo foi aprovado pelo conselho local de ética em pesquisa e todos os participantes forneceram consentimento informado por escrito para participação.

### Definições e desfechos

A classificação da NYHA é uma medida subjetiva, definida pelo médico, da limitação física de um paciente, variando desde nenhuma limitação durante atividade física normal (classe I) até sintomática em repouso (classe IV). A classe de Weber é derivada do consumo máximo de oxigênio durante o exercício (VO _2_ pico) medido durante o TCPE, sendo categorizada da seguinte maneira: classe A (VO _2_ pico > 20 ml/kg/min), B (16 a 20 ml/kg/min), C (10 a 16 ml/kg/min) e D (< 10 ml/kg/min). ^21^ No presente estudo, estratificamos as classes NYHA e Weber como “favoráveis” (NYHA I ou II; Weber A ou B) ou “adversas” (NYHA III ou IV; Weber C ou D). Foram classificados como “discordantes” pacientes com uma classe favorável e uma classe adversa (ou seja, NYHA I ou II com Weber C ou D; ou NYHA III ou IV com Weber A ou B). O desfecho primário do presente estudo foi a mortalidade por todas as causas. O estado vital foi avaliado prospectivamente por meio de registros eletrônicos de saúde e ligações telefônicas. Como parte da análise de sensibilidade, também estratificamos os pacientes em classificações favoráveis e adversas em relação à inclinação da relação entre ventilação minuto/produção de dióxido de carbono (VE/VCO _2_ ) e ao percentual atingido do VO _2_ pico predito (VO _2_ pp). A inclinação VE/VCO _2_ favorável foi definida como VE/VCO _2_ ≤ 36, e a inclinação VE/VCO _2_ adversa foi definida como VE/VCO _2_ > 36. O VO _2_ pp favorável foi definido como VO _2_ pp ≥ 50%, e o VO _2_ pp adverso foi definido como VO _2_ pp < 50%.

### Teste de exercício cardiopulmonar

A metodologia do TECP foi previamente relatada por nossa instituição ^22^ e segue recomendações previamente validadas. ^23^ O TECP foi realizado por cardiologistas experientes e treinados, utilizando protocolos institucionais padronizados. Resumidamente, o TCPE foi realizado em esteira (General Electric T-2100, GE Healthcare, EUA) com análise de gases respiração a respiração (Metalyzer 3B, Cortex, Leipzig, Alemanha ou Quark CPET, COSMED, Roma, Itália). Foi usado o teste de exercício máximo limitado por sintomas com um protocolo individualizado de rampa para produzir uma duração de exercício de 8 a 12 minutos limitada pela fadiga. O VO _2_ pico foi determinado pela medida mais alta de uma média móvel de 20 segundos dos valores respiração a respiração. A inclinação VE/VCO _2_ foi determinada por um modelo de regressão linear utilizando dados de toda a duração do teste. A inclinação da eficiência do consumo de oxigênio (OUES) foi derivada de um modelo semelhante, e as estimativas do ppVO _2_ utilizaram o algoritmo de Wasserman e Hansen, considerada a equação preferida para pacientes com IC. ^24^

### Análise estatística

As variáveis contínuas são apresentadas como mediana (percentis 25 e 75) porque um teste de Shapiro-Wilk indicou que todas as variáveis contínuas basais diferiram significativamente de uma distribuição normal. As variáveis categóricas são apresentadas como números absolutos e porcentagens. Foram utilizados testes de Kruskal-Wallis para comparar valores contínuos e testes de qui-quadrado para comparar proporções. Não foram utilizados testes *post hoc* . Para a análise principal do tempo até o óbito por todas as causas, os dois grupos de pacientes com classes NYHA e Weber discordantes (ou seja, classe NYHA favorável e classe Weber adversa; e classe NYHA adversa e classe Weber favorável) foram comparados usando um modelo de riscos proporcionais de Cox. O tempo até o óbito por todas as causas foi usado para produzir estimativas de Kaplan-Meier e analisado com estatísticas de log-rank. Além disso, para examinar visualmente a associação entre VO _2_ pico, classe NYHA e mortalidade, desenvolvemos um modelo multivariável de Cox para calcular as taxas de mortalidade previstas em 5 anos de acordo com VO _2_ pico e classe NYHA, ajustadas para idade e sexo na linha de base. Todas as análises foram realizadas usando R v4.0.2 (R Foundation for Statistical Computing, R Core Team, 2023). Um valor de p bilateral < 0,05 foi considerado estatisticamente significativo. O conjunto de dados utilizado para o presente manuscrito não está abertamente disponível, mas encorajamos os colegas a entrar em contato com o autor correspondente se tiverem interesse em colaborar.

## Resultados

### Características dos pacientes

As características clínicas dos 834 pacientes incluídos estão descritas na Tabela 1 . A mediana de idade foi de 57,1 anos; 42% (351) eram mulheres e a mediana da FEVE foi de 32,0%. O tempo mediano de acompanhamento foi de 3,1 anos (intervalo interquartil: 1,6 a 5,1). No geral, os pacientes estavam bem distribuídos entre as classes I, II e III da NYHA, com apenas 3% classificados como NYHA IV. Os pacientes nas classes menos graves de IC tinham maior probabilidade de ser do sexo masculino, de ter FEVE preservada (versus reduzida) e de usar inibidores da enzima conversora de angiotensina ou bloqueadores dos receptores de angiotensina.

**Tabela 1:** – Características basais

Características*	NYHA I (N=246)	NYHA II (N=362)	NYHA III (N=197)	NYHA IV (N=29)	Total (N=834)	Valor p
Idade, anos	57,1 (48,0-64,0)	56,8 (48,9-63,4)	58,2 (49,8-65,7)	56,1 (49,0-62,6)	57,1 (49,0-64,1)	0,346
Sexo feminino	89 (36,2%)	151 (41,7%)	98 (49,7%)	13 (44,8%)	351 (42,1%)	0,039
Índice de massa corporal, kg/m ^2^	26,1 (23,9-29,4)	28,2 (24,4-32,6)	27,7 (23,9-31,8)	26,6 (24,7-29,4)	27,4 (24,1-31,6)	0,004
Hipertensão	117 (47,6%)	200 (55,2%)	108 (54,8%)	12 (41,4%)	437 (52,4%)	0,15
Diabetes	65 (26,4%)	120 (33,1%)	72 (36,5%)	11 (37,9%)	268 (32,1%)	0,11
Fibrilação atrial	43 (17,5%)	71 (19,6%)	50 (25,4%)	8 (27,6%)	172 (20,6%)	0,15
**FEVE, %**	34,0 (25,0-45,3)	32,0 (25,0-45,0)	30,0 (23,0-38,0)	28,0 (20,0-53,0)	32,0 (25,0-43,0)	0,003
FEVE < 40%	150 (61,0%)	239 (66,0%)	149 (75,6%)	20 (69,0%)	558 (66,9%)	
FEVE 40,0% a 49,9%	42 (17,1%)	53 (14,6%)	19 (9,6%)	1 (3,4%)	115 (13,8%)	
FEVE ≥ 50%	52 (21,1%)	63 (17,4%)	24 (12,2%)	8 (27,6%)	147 (17,6%)	
Miocardiopatia isquêmica	59 (24,0%)	111 (30,7%)	72 (36,5%)	8 (27,6%)	250 (30,0%)	0,038
Uso de beta bloqueador	228 (92,7%)	345 (95,3%)	182 (92,4%)	26 (89,7%)	781 (93,6%)	0,34
Uso de IECA ou BRA	214 (87,0%)	304 (84,0%)	157 (79,7%)	16 (55,2%)	691 (82,9%)	<0,001
Uso de espironolactona	137 (55,7%)	229 (63,3%)	125 (63,5%)	14 (48,3%)	505 (60,6%)	0,11

Notas: *Os dados contínuos são apresentados como mediana (Q1-Q3); os dados categóricos são exibidos como N (%). Não foram utilizados inibidores do cotransportador sódio-glicose 2. BRA: bloqueadores dos receptores de angiotensina; FEVE: fração de ejeção do ventrículo esquerdo; IECA: inibidores da enzima conversora de angiotensina; NYHA: New York Heart Association. As medidas faltantes representaram 1,7% ou menos de cada variável.

### Características do teste de exercício cardiopulmonar

A Tabela 2 mostra a distribuição dos parâmetros do TECP por classe NYHA na linha de base. Nenhuma das variáveis contínuas apresentou distribuição normal. Os pacientes estavam bem distribuídos pelas classes de Weber, com aproximadamente um terço dos pacientes nas classes A, B e C, enquanto apenas 2% estavam na classe D. O VO _2_ pico foi significativamente menor em pacientes com classe NYHA mais alta, e os valores medianos variaram de 19,1 (classe I da NYHA) a 13,6 (classe IV da NYHA) ml/kg/min. O VO _2_ pp variou de 66,8% (NYHA I) a 48,1% (NYHA IV). A inclinação VE/VCO _2_ mediana de variou de 36,7 (NYHA I) a 49,8 (NYHA IV), e a OUES mediana variou de 1,40 (NYHA I) a 1,07 (NYHA IV). Para todas as variáveis, houve associação estatisticamente significativa entre parâmetros desfavoráveis do TECP e classe NYHA mais alta (p < 0,001).

**Tabela 2:** – Parâmetros do TECP por classe NYHA

Características	NYHA I (N=246)	NYHA II (N=362)	NYHA III (N=197)	NYHA IV (N=29)	Total (N=834)	Valor p
**VO**_**2**_ **pico, ml/kg/min**	19,1 (15,7-23,0)	17,4 (14,5-21,0)	15,3 (12,6-18,1)	13,6 (12,3-16,1)	17,2 (14,2-21,0)	<0,001
**Classe Weber**						<0,001
A (> 20 ml/kg/min)	107 (43%)	114 (31,5%)	27 (13,7%)	1 (3,4%)	249 (29,9%)	
B (16 a 20 ml/kg/min)	67 (27,2%)	112 (30,9%)	56 (28,4%)	7 (24,1%)	242 (29,0%)	
C (10 a 15,9 ml/kg/min)	71 (28,9%)	131 (36,2%)	101 (51,3%)	19 (65,5%)	322 (38,6%)	
D (< 10 ml/kg/min)	1 (0,4%)	5 (1,4%)	13 (6,6%)	2 (6,9%)	21 (2,5%)	
**Inclinação VE/VCO _**2**_**	36,7 (31,2-42,7)	37,2 (32,7-44,2)	41,0 (35,5-48,8)	49,8 (43,1-57,4)	38,1 (33,3-45,5)	<0,001
VE/VCO _2_ < 30	46 (18,7%)	57 (15,7%)	17 (8,6%)	1 (3,4%)	121 (14,5%)	
VE/VCO _2_ 30 a 35,9	48 (19,5%)	84 (23,2%)	66 (33,5%)	19 (65,5%)	217 (26,0%)	
VE/VCO _2_ 36 a 44,9	66 (26,8%)	92 (25,4%)	36 (18,3%)	0 (0%)	194 (23,3%)	
VE/VCO _2_ > 45	86 (35,0%)	129 (35,6%)	76 (38,6%)	9 (31,0%)	300 (36,0%)	
**OUES**	1,38 (1,11-1,88)	1,40 (1,01-1,76)	1,18 (0,861-1,56)	1,07 (0,827-1,19)	1,31 (0,990-1,72)	<0,001
OUES > 1,4	118 (48%)	178 (49,2%)	68 (34,5%)	3 (10,3%)	367 (44,0%)	
**VO**_**2**_ **pp**	0,67 (0,55-0,77)	0,64 (0,54-0,76)	0,58 (0,47-0,68)	0,48 (0,40-0,62)	0,63 (0,52-0,74)	<0,001
VO _2_ pp < 50%	38 (15,4%)	70 (19,3%)	60 (30,5%)	15 (51,7%)	183 (21,9%)	
VO _2_ pp ≥ 75%	72 (29,3%)	92 (25,4%)	30 (15,2%)	1 (3,4%)	195 (23,4%)	

Notas: *Os dados contínuos são apresentados como mediana (Q1-Q3); os dados categóricos são exibidos como N (%). NYHA: New York Heart Association; OUES: inclinação da eficiência do consumo de oxigênio; VE/VCO _2_: inclinação da relação entre ventilação minuto/produção de dióxido de carbono; VO _2_: consumo de oxigênio; VO _2_ pp: percentual atingido do VO _2_ pico predito. As medidas faltantes representaram 0,8% ou menos de cada variável.

### Valor prognóstico das classes NYHA e Weber

**Figura 1:**
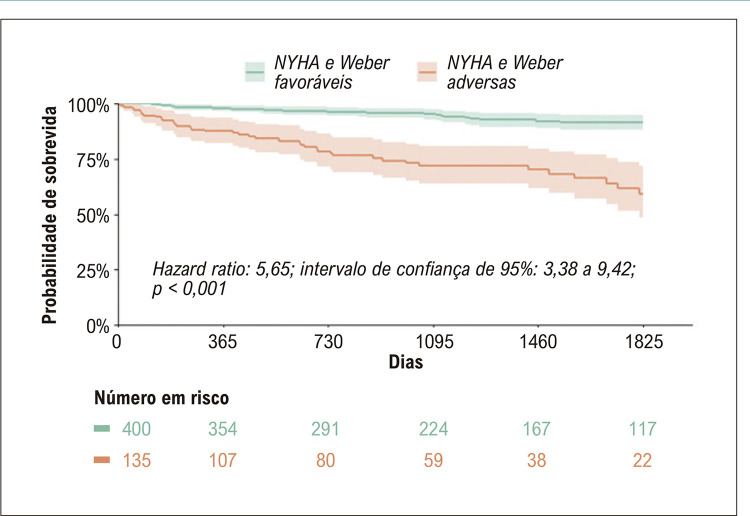
– Sobrevida global para pacientes com classes NYHA e Weber concordantes. Curva de Kaplan-Meier exibindo o tempo até o óbito por todas as causas. Classe NYHA favorável foi definida como NYHA I ou II; classe NYHA adversa foi definida como NYHA III ou IV. Classe Weber favorável foi definida como Weber A ou B; classe Weber adversa foi definida como Weber C ou D. Os pacientes com a mesma classificação (favorável ou adversa) em ambas as classificações foram classificados como concordantes e incluídos nesta análise. NYHA: New York Heart Association.

**Figura 2:**
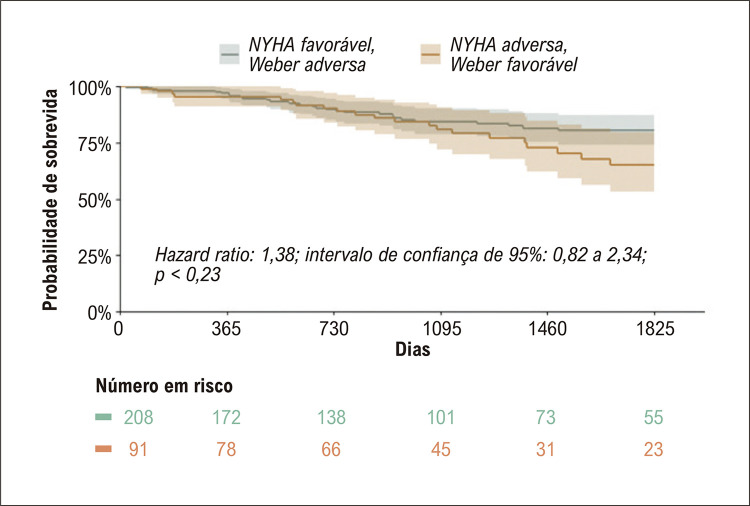
– Sobrevida global para pacientes com classes NYHA e Weber discordantes. Curva de Kaplan-Meier exibindo o tempo até o óbito por todas as causas. Classe NYHA favorável foi definida como NYHA I ou II; classe NYHA adversa foi definida como NYHA III ou IV. Classe Weber favorável foi definida como Weber A ou B; classe Weber adversa foi definida como Weber C ou D. Os pacientes com uma classe favorável e uma classe adversa foram classificados como discordantes e incluídos nesta análise. NYHA: New York Heart Association.

Um total de 64% (535) pacientes tinham classes NYHA e Weber concordantes (ou seja, NYHA I ou II com Weber A ou B; ou NYHA III ou IV com Weber C ou D). Entre aqueles com classes concordantes, os pacientes com ambas as classificações adversas apresentaram mortalidade significativamente maior ( Figura 1 ). Dos 299 pacientes com classificações discordantes, 208 (70%) tinham classe NYHA I ou II com classe C ou D de Weber, e 91 (30%) tinham classe III ou IV da NYHA com classe A ou B de Weber. Entre os dois grupos discordantes, os pacientes categorizados como tendo uma classe NYHA adversa e uma classe Weber favorável não tiveram taxas significativamente diferentes de mortalidade por todas as causas em comparação com pacientes que tinham uma classe NYHA favorável e uma classe Weber adversa ( Figura 2 ). Os achados foram mantidos ao comparar medidas alternativas de TECP, como inclinação VE/VCO _2_ ( *hazard ratio* [HR]: 1,11; intervalo de confiança de 95%: 0,58 a 2,14; p = 0,74; Figura Suplementar 1) e VO _2_ pp (HR: 0,98; intervalo de confiança de 95%: 0,57 a 1,69; Figura Suplementar 2).

**Figura 3:**
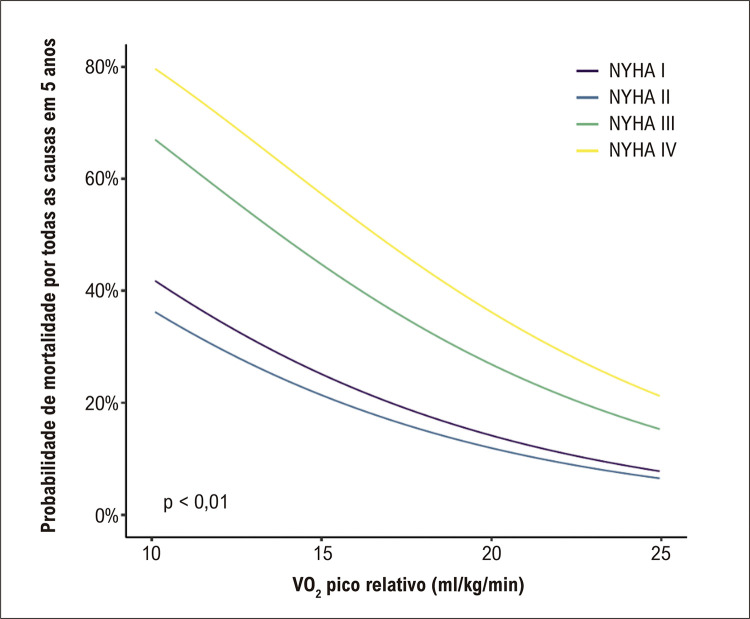
– Probabilidade prevista de mortalidade em 5 anos por classe NYHA e VO 2 pico. Probabilidade prevista de mortalidade para um paciente masculino de 57,2 anos de idade. NYHA: New York Heart Association; VO 2 : consumo de oxigênio.

No modelo multivariável, tanto classe NYHA mais alta (HR: 1,55 por aumento de unidade; intervalo de confiança de 95%: 1,26 a 1,92; p < 0,001) quanto VO _2_ pico mais baixo (HR: 1,47 por diminuição de 3 ml/kg/min; intervalo de confiança de 95%: 1,28 a 1,70; p < 0,001) previram independentemente a mortalidade por todas as causas. Quando a classificação da NYHA foi analisada como variável categórica, a diferença entre as classes NYHA I e II foi de magnitude menor e não estatisticamente significativa (HR: 0,83 para NYHA II versus I; intervalo de confiança de 95%: 0,51 a 1,36; p = 0,46). A Figura 3 mostra a taxa de mortalidade prevista em 5 anos de acordo com a classe NYHA e o VO _2_ pico na linha de base. Cada variável mostrou ter um valor preditivo independente. Por exemplo, para pacientes de qualquer classe da NYHA, as taxas de mortalidade em 5 anos foram mais de 2 vezes maiores se o VO _2_ pico fosse de 12 ml/kg/min em vez de 20 ml/kg/min. Por outro lado, para pacientes com um determinado VO _2_ pico, a taxa de mortalidade em 5 anos aproximadamente dobrou se fossem classificados como NYHA III em vez de NYHA II.

## Discussão

Em uma grande coorte de pacientes com IC submetidos a TECP, tanto a classificação NYHA quanto o VO _2_ pico foram preditores independentes de mortalidade por todas as causas. Os pacientes que tinham classes NYHA mais altas apresentaram resultados consistentemente piores no TECP. Além disso, os pacientes com uma classe NYHA favorável e uma classe Weber adversa tiveram um risco intermediário de mortalidade por todas as causas que não foi significativamente diferente dos pacientes com uma classe NYHA adversa e uma classe Weber favorável.

Estudos anteriores analisaram a importância prognóstica da classificação da NYHA. Muntwyler et al. mostraram que a classe NYHA foi um fator prognóstico independente na análise multivariada, com mortalidade em 1 ano variando de 7,1% em pacientes com NYHA II a 28,0% naqueles com NYHA IV. ^6^ A classificação da NYHA também permaneceu um preditor potente de mortalidade em pelo menos 10 anos. ^7^ Vários outros estudos, no entanto, sugeriram que a classificação da NYHA pode ser um marcador de prognóstico não confiável em nível individual. Caraballo et al. mostraram heterogeneidade significativa do risco de mortalidade em pacientes com NYHA II e III entre estudos, sugerindo que a implicação prognóstica da classificação NYHA depende em grande parte do risco basal do paciente sendo avaliado. ^13^ Mais recentemente, Blacher et al. mostraram sobreposição significativa em várias métricas entre pacientes com NYHA I e II, sugerindo que a classe NYHA, por si só, pode ser um discriminador insuficiente de pacientes individuais com IC leve. ^11^ Também foi estudado se as mudanças na classe NYHA ao longo do tempo podem prever o prognóstico. Greene et al. mostraram que melhorias na classe NYHA não levaram a melhores desfechos em pacientes com IC, enquanto a melhora na pontuação geral do Kansas City Cardiomyopathy Questionnaire Overall Summary Score foi correlacionada com melhora no prognóstico. ^25^ Rohde et al. sugeriram que mudanças na classe NYHA ao longo do tempo podem ter valor preditivo limitado, particularmente na IC leve. ^12^

O TECP tem sido cada vez mais proposto como forma de melhorar a avaliação prognóstica em pacientes com IC, oferecendo métricas objetivas e reprodutíveis. ^10 , 15^ O TECP tem sido utilizado como ferramenta para auxiliar na tomada de decisão sobre transplante cardíaco há mais de 3 décadas, ^18^ dada a sua confiabilidade na distinção de risco entre pacientes com IC grave. Também houve apelos para a inclusão do TECP como parte dos critérios de inscrição e de desfecho em ensaios de IC já em 1988. ^15^ Foi demonstrado que muitas métricas do TECP têm implicações prognósticas, incluindo VO _2_ pico, OUES, inclinação VE/VCO _2_ , pressão de CO _2_ no final de expiração em repouso e ventilação oscilatória de exercício. ^18 , 19 , 26 - 28^

A maioria dos estudos prognósticos teve como foco a classificação da NYHA ou o TECP, mas raramente em ambos. Isso deixa os médicos inseguros sobre como interpretar as informações derivadas das avaliações simultâneas da NYHA e do TECP. Isso é especialmente importante quando se deparam com informações conflitantes, por exemplo, um paciente classificado em classe NYHA avançada com TECP apresentando classificação de Weber favorável (classe A ou B). Nosso estudo visou combinar essas avaliações para refinar a avaliação prognóstica em pacientes com IC. Na presente análise de uma grande coorte de pacientes com IC submetidos a TECP, tanto a classificação da NYHA quanto o VO _2_ pico foram preditivos de mortalidade por todas as causas após ajuste para idade e sexo. Em todas as classes da NYHA, as diminuições no VO _2_ pico foram associadas ao aumento da mortalidade; da mesma forma, em todo o espectro do VO _2_ pico, os incrementos na classificação da NYHA também foram associados ao aumento da mortalidade. A exceção notável foi a falta de uma diferença significativa entre as classes I e II da NYHA, em conformidade com estudos anteriores. ^9 , 11 , 12^ Este achado é crítico porque os pacientes classificados como NYHA I têm sido excluídos dos ensaios clínicos de IC com base na suposição de que eles constituem um grupo uniformemente de baixo risco. Os pacientes com NYHA I são, portanto, inelegíveis para diversas terapias de prolongamento da vida que estão bem estabelecidas para pacientes com IC na classe NYHA II e acima. ^3 , 4^ Além disso, visamos analisar o valor prognóstico de ambas as classificações quando os pacientes apresentavam resultados do TECP que aparentemente conflitavam com a classe NYHA atribuída pelo médico. Não encontramos diferença significativa na mortalidade por todas as causas entre pacientes com classes discordantes (NYHA I ou II com Weber C ou D versus NYHA III ou IV com Weber A ou B), sugerindo que ambas as métricas da NYHA e do TECP são variáveis prognósticas complementares. Os pacientes com classes discordantes apresentaram prognóstico intermediário em comparação com aqueles com classificações concordantes favoráveis (NYHA I ou II e Weber A ou B) ou concordantes adversas (NYHA III ou IV e Weber C ou D). Nossos achados foram consistentes nas análises de sensibilidade usando a inclinação VE/VCO _2_ e VO _2_ pp em vez do VO _2_ pico para determinar classes conflitantes. Nossos resultados estão em conformidade com um estudo anterior de Ritt et al. que demonstrou associação entre as classes NYHA e Weber, embora com baixa concordância entre elas. ^29^

Nosso estudo teve algumas limitações que merecem consideração. Primeiro, embora normalmente realizado dentro de semanas, período em que não se espera que o estado funcional de um paciente com IC mude, o momento exato entre a definição da NYHA e o TECP não foi registrado. Em segundo lugar, o presente estudo foi retrospectivo e incluiu uma avaliação única da classe NYHA e os resultados não podem ser extrapolados para a variação da classe da NYHA ao longo do tempo. Terceiro, para a análise longitudinal, não está claro como os resultados do TECP foram usados para orientar as decisões terapêuticas. Além disso, não estudamos o impacto de TCPEs repetidos nesta população. Finalmente, o período do estudo abrange mais de uma década e a prática clínica pode ter mudado ao longo desse período.

## Conclusão

A classificação da NYHA e os parâmetros do TECP fornecem informações prognósticas complementares que são mais precisas do que o uso de qualquer um dos dois isoladamente. O TCPE pode ser uma ferramenta valiosa para discriminar o risco em pacientes com IC em todas as classes da NYHA, particularmente para aqueles nas classes NYHA I e II.
