# Supplementary material for: Papel Incremental da Classificação da New York Heart Association e dos Índices do Teste de Exercício Cardiopulmonar para Prognóstico na Insuficiência Cardíaca: um Estudo de Coorte
Source: Arq Bras Cardiol. 2023 Dec 6;120(11):e20230077. [Article in Portuguese] doi: 10.36660/abc.20230077 (PMC10773458; doi:10.36660/abc.20230077)
Supplement: Translation

# Translation: Incremental Role of New York Heart Association Class and Cardiopulmonary Exercise Test Indices for Prognostication in Heart Failure: A Cohort Study

**Authors:** Pedro Henrique de Borba Engster, André Zimerman, Thomas Schaan, Marina S. Borges, Gabriel Souza, Giovanni Donelli Costa, Luis Eduardo Rohde, Anderson Donelli da Silveira

**Affiliations:** 1 Brasil Universidade Federal do Rio Grande do Sul – FAMED, Porto Alegre, RS – Brasil; 2 Brasil Hospital de Clínicas de Porto Alegre, Porto Alegre, RS – Brasil; 3 EUA Harvard Medical School, Boston, Massachusetts – EUA; 4 Brasil Hospital Moinhos de Vento – Cardiologia, Porto Alegre, RS – Brasil

**Keywords:** Heart Failure, Prognosis, Exercise Test

## Abstract

**Background:**

The accuracy of the New York Heart Association (NYHA) classification to assess prognosis may be limited compared with objective cardiopulmonary exercise test (CPET) parameters in heart failure (HF).

**Objective:**

To investigate the prognostic value of the NYHA classification in addition to Weber class.

**Methods:**

Adult outpatients with HF undergoing CPET in a Brazilian tertiary care center were included. The physician-assigned NYHA class and the CPET-derived Weber class were stratified into “favorable” (NYHA I or II; Weber A or B) or “adverse” (NYHA III or IV; Weber C or D). Patients with one favorable class and one adverse class were defined as “discordant.” The primary endpoint was time to all-cause mortality. A 2-sided p value < 0.05 was considered statistically significant.

**Results:**

A total of 834 patients were included. Median age was 57 years; 42% (351) were female, and median left ventricular ejection fraction was 32%. Among patients with concordant NYHA and Weber classes, those with adverse NYHA and Weber classes had significantly higher all-cause mortality compared to those with favorable classes (hazard ratio [HR]: 5.65; 95% confidence interval [CI]: 3.38 to 9.42). Among patients with discordant classes, there was no significant difference in all-cause mortality (HR: 1.38; 95% CI: 0.82 to 2.34). In the multivariable model, increments in NYHA class (HR: 1.55 per class increase; 95% CI: 1.26 to 1.92) and reductions in peak VO _2_ (HR: 1.47 per 3 ml/kg/min decrease; 95% CI: 1.28 to 1.70) significantly predicted mortality.

**Conclusions:**

Physician-assigned NYHA class and objective CPET measures provide complementary prognostic information for patients with HF.

## Introduction

Heart failure (HF) is one of the leading causes of morbidity and mortality worldwide, affecting over 64 million people. ^1^ One of the cornerstones of HF management is the definition of a patient’s New York Heart Association (NYHA) classification, proposed in 1921 to measure functional impairment. ^2^ This subjective measurement has been widely used as an inclusion criterion for clinical protocols. Patients considered asymptomatic at ordinary physical activity (namely, NYHA class I) have been systematically excluded from HF trials. Consequently, clinical guidelines frequently use an NYHA class cutoff to determine eligibility for treatments such as mineralocorticoid receptor antagonists, sodium-glucose cotransporter-2 inhibitors, and cardiac resynchronization therapy. ^3 - 5^ The NYHA classification is an established, powerful predictor of HF prognosis at a group level. ^6 - 8^ Recent studies, however, have questioned the reproducibility of the NYHA classification and its ability to discriminate the prognosis of patients with HF at the individual level. ^9 - 14^

These limitations have encouraged efforts to attain more accurate and reproducible parameters of functional capacity in patients with HF, ranging from structured questionnaires to objective measurements of functional capacity, such as cardiopulmonary exercise testing (CPET). ^10 , 15^ CPET is a non-invasive method to analyze cardiopulmonary fitness and establish functional status. Currently, CPET is used to assess HF severity, monitor disease progression, and determine eligibility for heart transplantation. ^16 - 18^

For patients with HF, both the physician-assigned NYHA class and the objective CPET parameters have been shown to be independent prognostic factors. ^7 , 19 , 20^ However, as two surrogates of functional capacity, one subjective and one objective, their combined ability for prognostication is less clear. For example, for patients who have undergone a CPET, it is plausible that the NYHA classification lacks additional prognostic value. In the current analysis, we investigated the interplay of CPET-derived indices and NYHA class to refine prognostic assessment in patients with HF, particularly when CPET and NYHA class depicted conflicting results.

## Methods

### Patients and study design

This cohort study included consecutive patients with HF who underwent a CPET in a tertiary care hospital in Brazil between January 2008 and November 2020. The first CPET of each patient was included in this analysis. NYHA class was determined immediately before CPET or in the previous outpatient visit. Eligible patients were 16 years or older with documented HF, diagnosed by clinical, laboratory, and echocardiographic criteria. ^3^ Subjects had to be clinically stable prior to CPET and using optimal medical therapy. There were no left ventricular ejection fraction (LVEF) eligibility criteria, i.e., patients with reduced, mildly reduced, and preserved LVEF were eligible for enrollment. Patients who were unable to perform a CPET were excluded. This study was approved by the local research ethics board, and all participants provided written informed consent for participation.

### Definitions and endpoints

The NYHA classification is a subjective, physician-defined measure of a patient’s physical limitation, ranging from no limitation at ordinary physical activity (class I) to symptomatic at rest (class IV). The Weber class is derived from the maximum oxygen consumption during exercise (peak VO _2_ ) measured during CPET and is categorized into class A (peak VO _2_ > 20 ml/kg/min), B (16 to 20 ml/kg/min), C (10 to 16 ml/kg/min), and D (< 10 ml/kg/min). ^21^ In this study, we stratified NYHA and Weber classes into “favorable” (NYHA I or II; Weber A or B) or “adverse” (NYHA III or IV; Weber C or D). Subjects with one favorable class and one adverse class (i.e., NYHA I or II with Weber C or D, or NYHA III or IV with Weber A or B) were classified as “discordant.” The primary endpoint of this study was all-cause mortality. Vital status was prospectively evaluated using electronic health records and telephone calls. As part of a sensitivity analysis, we also stratified patients into favorable and adverse classifications regarding minute ventilation/carbon dioxide production (VE/VCO _2_ ) slope and percent-predicted peak VO _2_ (ppVO _2_ ). Favorable VE/VCO _2_ slope was defined as VE/VCO _2_ ≤ 36, and adverse VE/VCO _2_ slope was defined as VE/VCO _2_ > 36. Favorable ppVO _2_ was defined as ppVO _2_ ≥ 50%, and adverse ppVO _2_ was defined as ppVO _2_ < 50%.

### Cardiopulmonary exercise testing

CPET methodology has been previously reported by our institution, ^22^ and it follows previously validated recommendations. ^23^ CPET was conducted by experienced and trained cardiologists using standardized institutional protocols. In brief, CPET was performed on a treadmill (General Electric T-2100, GE Healthcare, USA) with breath-by-breath gas analysis (Metalyzer 3B, Cortex, Leipzig, Germany or Quark CPET, COSMED, Rome, Italy). Symptom-limited maximal exercise testing with an individualized ramp protocol was used to yield fatigue-limited exercise duration of 8 to 12 minutes. Peak VO _2_ was determined by the highest measure of a 20-second rolling average of breath-by-breath values. VE/VCO _2_ slope was determined by a linear regression model using data from the entire duration of the test. Oxygen uptake efficiency slope (OUES) was derived from a similar model, and ppVO _2_ estimations used Wasserman and Hansen’s algorithm, considered the preferred equation for patients with HF. ^24^

### Statistical analysis

Continuous variables are displayed as median (25 ^th^ and 75 ^th^ percentiles), as a Shapiro-Wilk test indicated that all continuous baseline variables significantly differed from a normal distribution. Categorical variables are displayed as absolute numbers and percentages. Kruskal-Wallis tests were used to compare continuous values, and chi-square tests were used to compare proportions. No *post hoc* tests were used. For the main analysis of time to all-cause death, the two groups of subjects with discordant NYHA and Weber classes (i.e., favorable NYHA and adverse Weber class, and adverse NYHA and favorable Weber class) were compared using a Cox proportional hazards model. Time to all-cause death was used to produce Kaplan-Meier estimates and analyzed with log-rank statistics. Furthermore, to visually examine the association between peak VO _2_ , NYHA class, and mortality, we developed a multivariable Cox model to compute the predicted 5-year mortality rates according to peak VO _2_ and NYHA class, adjusted for age and sex at baseline. All analyses were performed using R v4.0.2 (R Foundation for Statistical Computing, R Core Team, 2023). A 2-sided p value < 0.05 was considered statistically significant. The dataset used for this manuscript is not openly available, but we encourage colleagues to contact the corresponding author if they are interested in collaborating.

## Results

### Patient characteristics

The clinical characteristics of the 834 patients included are described in Table 1 . Median age was 57.1 years; 42% (351) were female, and median LVEF was 32.0%. Median follow-up time was 3.1 years (interquartile range: 1.6 to 5.1). Overall, patients were well distributed between NYHA classes I, II, and III, with only 3% classified as NYHA IV. Patients in milder HF classes were more likely to be male, to have preserved (versus reduced) LVEF, and to be using angiotensin-converting enzyme inhibitors or angiotensin receptor blockers.

**Table 1:** – Baseline characteristics

Caracteristics*	NYHA I (N=246)	NYHA II (N=362)	NYHA III (N=197)	NYHA IV (N=29)	Overall (N=834)	p value
Age, years	57.1 (48.0-64.0)	56.8 (48.9-63.4)	58.2 (49.8-65.7)	56.1 (49.0-62.6)	57.1 (49.0-64.1)	0.346
Female sex	89 (36.2%)	151 (41.7%)	98 (49.7%)	13 (44.8%)	351 (42.1%)	0.039
Body mass index, kg/m ^2^	26.1 (23.9-29.4)	28.2 (24.4-32.6)	27.7 (23.9-31.8)	26.6 (24.7-29.4)	27.4 (24.1-31.6)	0.004
Hypertension	117 (47.6%)	200 (55.2%)	108 (54.8%)	12 (41.4%)	437 (52.4%)	0.15
Diabetes	65 (26.4%)	120 (33.1%)	72 (36.5%)	11 (37.9%)	268 (32.1%)	0.11
Atrial fibrillation	43 (17.5%)	71 (19.6%)	50 (25.4%)	8 (27.6%)	172 (20.6%)	0.15
**LVEF, %**	34.0 (25.0-45.3)	32.0 (25.0-45.0)	30.0 (23.0-38.0)	28.0 (20.0-53.0)	32.0 (25.0-43.0)	0.003
LVEF < 40%	150 (61.0%)	239 (66.0%)	149 (75.6%)	20 (69.0%)	558 (66.9%)	
LVEF 40.0% to 49.9%	42 (17.1%)	53 (14.6%)	19 (9.6%)	1 (3.4%)	115 (13.8%)	
LVEF ≥ 50%	52 (21.1%)	63 (17.4%)	24 (12.2%)	8 (27.6%)	147 (17.6%)	
Ischemic cardiomyopathy	59 (24.0%)	111 (30.7%)	72 (36.5%)	8 (27.6%)	250 (30.0%)	0.038
Beta blocker use	228 (92.7%)	345 (95.3%)	182 (92.4%)	26 (89.7%)	781 (93.6%)	0.34
ACEI or ARB use	214 (87.0%)	304 (84.0%)	157 (79.7%)	16 (55.2%)	691 (82.9%)	<0.001
Spironolactone use	137 (55.7%)	229 (63.3%)	125 (63.5%)	14 (48.3%)	505 (60.6%)	0.11

* Continuous data are displayed as median (Q1-Q3); categorical data are displayed as N (%). Sodium-glucose cotransporter-2 inhibitors were not used. ACEI angiotensin converting enzyme inhibitor; ARB: angiotensin receptor blocker; LVEF: left ventricular ejection fraction; NYHA: New York Heart Association. Missing measurements accounted for 1.7% or less of each variable.

### Cardiopulmonary exercise test characteristics

Table 2 illustrates the distribution of CPET parameters by baseline NYHA class. None of the continuous variables were normally distributed. Patients were well distributed across Weber classes, with approximately one third of patients in classes A, B, and C, while only 2% were in class D. Peak VO _2_ was significantly lower in patients with higher NYHA class, and median values ranged from 19.1 (NYHA class I) to 13.6 (NYHA class IV) ml/kg/min. ppVO _2_ varied from 66.8% (NYHA I) to 48.1% (NYHA IV). Median VE/VCO _2_ slope ranged from 36.7 (NYHA I) to 49.8 (NYHA IV), and median OUES ranged from 1.40 (NYHA I) to 1.07 (NYHA IV). Across all variables, there was a statistically significant association between unfavorable CPET parameters and higher NYHA class (p < 0.001).

**Table 2:** – CPET parameters by NYHA class

Caracteristics	NYHA I (N=246)	NYHA II (N=362)	NYHA III (N=197)	NYHA IV (N=29)	Overall (N=834)	p value
**Peak VO**_**2**_ **, ml/kg/min**	19.1 (15.7-23.0)	17.4 (14.5-21.0)	15.3 (12.6-18.1)	13.6 (12.3-16.1)	17.2 (14.2-21.0)	<0.001
**Weber class**						<0.001
A (> 20 ml/kg/min)	107 (43%)	114 (31.5%)	27 (13.7%)	1 (3.4%)	249 (29.9%)	
B (16 to 20 ml/kg/min)	67 (27.2%)	112 (30.9%)	56 (28.4%)	7 (24.1%)	242 (29.0%)	
C (10 to 15.9 ml/kg/min)	71 (28.9%)	131 (36.2%)	101 (51.3%)	19 (65.5%)	322 (38.6%)	
D (< 10 ml/kg/min)	1 (0.4%)	5 (1.4%)	13 (6.6%)	2 (6.9%)	21 (2.5%)	
**VE/VCO _**2**_ slope**	36.7 (31.2-42.7)	37.2 (32.7-44.2)	41.0 (35.5-48.8)	49.8 (43.1-57.4)	38.1 (33.3-45.5)	<0.001
VE/VCO _2_ < 30	46 (18.7%)	57 (15.7%)	17 (8.6%)	1 (3.4%)	121 (14.5%)	
VE/VCO _2_ 30 to 35.9	48 (19.5%)	84 (23.2%)	66 (33.5%)	19 (65.5%)	217 (26.0%)	
VE/VCO _2_ 36 to 44.9	66 (26.8%)	92 (25.4%)	36 (18.3%)	0 (0%)	194 (23.3%)	
VE/VCO _2_ > 45	86 (35.0%)	129 (35.6%)	76 (38.6%)	9 (31.0%)	300 (36.0%)	
**OUES**	1.38 (1.11-1.88)	1.40 (1.01-1.76)	1.18 (0.861-1.56)	1.07 (0.827-1.19)	1.31 (0.990-1.72)	<0.001
OUES > 1.4	118 (48%)	178 (49.2%)	68 (34.5%)	3 (10.3%)	367 (44.0%)	
**Percent-predicted peak VO**_**2**_	0.67 (0.55-0.77)	0.64 (0.54-0.76)	0.58 (0.47-0.68)	0.48 (0.40-0.62)	0.63 (0.52-0.74)	<0.001
Percent-predicted peak VO _2_ < 50%	38 (15.4%)	70 (19.3%)	60 (30.5%)	15 (51.7%)	183 (21.9%)	
Percent-predicted peak VO _2_ ≥ 75%	72 (29.3%)	92 (25.4%)	30 (15.2%)	1 (3.4%)	195 (23.4%)	

*Continuous data are displayed as median (Q1-Q3); categorical data are displayed as N (%). NYHA: New York Heart Association; OUES: oxygen uptake efficiency slope; VE/VCO _2_: minute ventilation/carbon dioxide output slope; VO _2_: oxygen consumption. Missing measurements accounted for 0.8% or less of each variable.

### Prognostic value of NYHA and Weber classes

A total of 64% (535) patients had concordant NYHA and Weber classes (i.e., NYHA I or II with Weber A or B, or NYHA III or IV with Weber C or D). Among those with concordant classes, patients with both adverse classifications had significantly higher mortality ( Figure 1 ). Of the 299 patients with discordant classifications, 208 (70%) had NYHA class I or II with Weber class C or D, and 91 (30%) had NYHA class III or IV with Weber class A or B. Among the two discordant groups, patients categorized as having an adverse NYHA class and a favorable Weber class did not have significantly different rates of all-cause mortality compared with patients who had a favorable NYHA class and an adverse Weber class ( Figure 2 ). Findings were maintained when comparing alternative CPET measures, such as VE/VCO _2_ slope (hazard ratio [HR]: 1.11; 95% confidence interval [CI]: 0.58 to 2.14; p = 0.74; Supplementary Figure 1 ) and ppVO _2_ (HR: 0.98; 95% CI: 0.57 to 1.69; Supplementary Figure 2 ).

**Figure 1:** – Overall survival for patients with concordant NYHA and Weber classes. Notes: Kaplan-Meier curve displaying time to all-cause death. Favorable NYHA class was defined as NYHA I or II; adverse NYHA class was defined as NYHA III or IV. Favorable Weber class was defined as Weber A or B; adverse Weber class was defined as Weber C or D. Patients with the same classification (either favorable or adverse) in both classes were classified as concordant and included in this analysis. CI: confidence interval; HR: hazard ratio; NYHA: New York Heart Association.

**Figure 2:** – Overall survival for patients with discordant NYHA and Weber classes. Notes: Kaplan-Meier curve displaying time to all-cause death. Favorable NYHA class was defined as NYHA I or II; adverse NYHA class was defined as NYHA III or IV. Favorable Weber class was defined as Weber A or B; adverse Weber class was defined as Weber C or D. Patients with one favorable class and one adverse class were classified as discordant and included in this analysis. CI: confidence interval; HR: hazard ratio; NYHA: New York Heart Association.

In the multivariable model, both higher NYHA class (HR: 1.55 per unit increase; 95% CI: 1.26 to 1.92; p < 0.001) and lower peak VO _2_ (HR: 1.47 per 3 ml/kg/min decrease; 95% CI: 1.28 to 1.70; p < 0.001) independently predicted all-cause mortality. When the NYHA classification was analyzed as a categorical variable, the difference between NYHA classes I and II was minor in magnitude and not statistically significant (HR: 0.83 for NYHA II versus I; 95% CI: 0.51 to 1.36; p = 0.46). Figure 3 displays the predicted 5-year mortality rate according to baseline NYHA class and peak VO _2_ . Each variable was shown to have an independent predictive value. For example, for patients within any NYHA class, 5-year mortality rates were more than 2-fold higher if the peak VO _2_ was 12 ml/kg/min instead of 20 ml/kg/min. Conversely, for patients with a certain peak VO _2_ , the 5-year mortality rate approximately doubled if they were classified as NYHA III instead of NYHA II.

**Figure 3:** – Predicted probability of mortality at 5 years by NYHA class and peak VO 2 . Predicted probability of mortality for a male patient of age 57.2 years. NYHA: New York Heart Association; VO 2 : oxygen consumption.

## Discussion

In a large cohort of patients with HF undergoing CPET, both NYHA classification and peak VO _2_ were independent predictors of all-cause mortality. Patients who exhibited higher NYHA classes consistently presented worse results in the CPET. Furthermore, patients with a favorable NYHA class and an adverse Weber class had an intermediate risk of all-cause mortality that was not significantly different from patients with an adverse NYHA class and a favorable Weber class.

Previous studies have analyzed the prognostic importance of the NYHA classification. Muntwyler et al. showed NYHA class to be an independent prognostic factor in multivariable analysis, with 1-year mortality ranging from 7.1% in patients with NYHA II to 28.0% in those with NYHA IV. ^6^ NYHA classification also remained a powerful predictor of mortality for at least 10 years. ^7^ Several other studies, however, have suggested that NYHA classification might be an unreliable marker of prognosis on an individual level. Caraballo et al. showed significant heterogeneity of mortality risk in NYHA II and III patients across studies, suggesting that the prognostic implication of the NYHA classification is largely dependent on the baseline risk of the patient being assessed. ^13^ More recently, Blacher et al. showed significant overlap in several metrics between NYHA I and II patients, suggesting that the NYHA class, by itself, may be an insufficient discriminator of individual patients with mild HF. ^11^ The question of whether changes in NYHA class over time can predict prognosis has been studied as well. Greene et al. showed that improvements in NYHA class did not lead to better outcomes in patients with HF, while improvement in Kansas City Cardiomyopathy Questionnaire Overall Summary Score was correlated with improved prognosis. ^25^ Rohde et al. suggested that changes in NYHA class over time may have limited predictive value, particularly in mild HF. ^12^

CPET has been increasingly proposed as a way to improve prognostic assessment in patients with HF by offering objective and reproducible metrics. ^10 , 15^ CPET has been used as a tool to aid in cardiac transplantation decision-making for over three decades, ^18^ given its reliability in distinguishing risk among patients with severe HF. There have also been calls for the inclusion of CPET as part of the enrollment and endpoint criteria in HF trials as early as 1988. ^15^ Many CPET metrics have been shown to have prognostic implications, including peak VO _2_ , OUES, VE/VCO _2_ slope, resting end-tidal CO _2_ pressure, and exercise oscillatory ventilation. ^18 , 19 , 26 - 28^

Most prognostic studies have focused either on NYHA classification or CPET, but rarely on both. This leaves clinicians unsure of how to interpret the information derived from simultaneous NYHA and CPET assessments. This is especially important when they are faced with conflicting information, such as a patient categorized in an advanced NYHA class with CPET showing favorable Weber classification (class A or B). Our study aimed to combine these assessments to refine the prognostic evaluation in patients with HF. In this analysis of a large cohort of patients with HF undergoing CPET, both NYHA classification and peak VO _2_ were predictive of all-cause mortality after adjusting for age and sex. Across all NYHA classes, decreases in peak VO _2_ were associated with increased mortality; likewise, across the spectrum of peak VO _2_ , increments in NYHA classification were also linked to increased mortality. The notable exception was the lack of a significant difference between NYHA classes I and II, in conformity with prior studies. ^9 , 11 , 12^ This finding is critical because patients classified as NYHA I have been excluded from HF clinical trials based on the assumption that they constitute a uniformly low-risk group, and NYHA I patients are thus ineligible for several life-prolonging therapies that are well established for patients with HF in NYHA class II and above. ^3 , 4^ Furthermore, we sought to analyze the prognostic value of both classifications when patients had CPET results that apparently conflicted with their physician-assigned NYHA class. We found no significant difference in all-cause mortality between patients with discordant classes (NYHA I or II with Weber C or D versus NYHA III or IV with Weber A or B), suggesting that both NYHA and CPET metrics are complementary prognostic variables. Patients with discordant classes displayed an intermediate prognosis compared to those with concordant favorable (NYHA I or II and Weber A or B) or concordant adverse (NYHA III or IV and Weber C or D) classifications. Our findings were consistent in sensitivity analyses using VE/VCO _2_ slope and ppVO _2_ instead of peak VO _2_ to determine conflicting classes. Our results are in conformity with a previous study by Ritt et al. that demonstrated an association between NYHA and Weber classes, albeit with low concordance between them. ^29^

Our study had limitations that merit consideration. First, although ordinarily performed within weeks, a period in which the functional status of a patient with HF is not expected to shift, the exact timing between NYHA determination and the CPET was not recorded. Second, this study was retrospective and included a one-time NYHA assessment, and results cannot be extrapolated to NYHA class variation over time. Third, for the longitudinal analysis, it is unclear how CPET findings were used to guide therapeutic decisions. Furthermore, we did not study the impact of repeated CPETs in this population. Finally, the study time frame spans over a decade, and clinical practice might have shifted over that time.

## Conclusion

NYHA classification and CPET parameters provide complementary prognostic information that is more accurate than using either alone. CPET may be a valuable tool to discriminate risk in patients with HF across all NYHA classes, particularly for those in NYHA classes I and II.
